# Circular SAW Resonators: Influence of Sensitive Element Dimensions on Strength Characteristics and First Experimental Samples †

**DOI:** 10.3390/s24144584

**Published:** 2024-07-15

**Authors:** Sergey Yu. Shevchenko, Denis A. Mikhailenko, Alexander S. Kukaev, Vladimir Yu. Venediktov

**Affiliations:** Department of Laser Measuring and Navigation Systems, Faculty of Information Measurement and Biotechnical Systems, Saint Petersburg Electrotechnical University (LETI), Popova Str., h. 5, 197376 Saint Petersburg, Russia; kratosloaded@mail.ru (D.A.M.); askukaev@gmail.com (A.S.K.); vlad.venediktov@mail.ru (V.Y.V.)

**Keywords:** surface acoustic waves, microelectromechanical systems, micromechanical accelerometer, sensitive element, resonator, interdigital transducer

## Abstract

In preceding research endeavors, the frequency characteristics of a ring resonator on surface acoustic waves made of various materials were studied. Investigations encompassed fixation techniques within the housing, the impact of external variables on these components, and the most efficient configuration of the interdigital transducer within the ring resonator to curtail bandwidth. This current study is dedicated to investigating the correlation between sensitivity and the highest measurable acceleration concerning the dimensions of these sensitive elements. Furthermore, it involves assessing the attributes of produced experimental samples to verify the simulation results. The results obtained represent the possibility of creating a micromechanical accelerometer that can be used in the automotive industry as a g-sensor shock, as well as in industries where the numerical value of high overloads is required.

## 1. Introduction

Microelectromechanical systems (MEMS) represent a microelectronics innovation that has gained prominence due to the global trend of miniaturization. MEMS entails the reduction in mechanical systems to micro-scale dimensions and their integration with electrical circuits. This integration yields a physical apparatus where all elements are interconnected, harmonized, and designed to perform specific functions.

The main advantages of MEMS are its minimal size due to the placement of all components on a single substrate, economic efficiency due to the automation of the manufacturing process of mass production and serial production of products, low power consumption at the level of several watts due to the compactness of technological solutions, and increased measurement frequency.

Despite many advantages associated with microelectromechanical systems, they also exhibit drawbacks such as low precision and mechanical strength coming from high vulnerability to external influences.

Taking into account all of these factors, microelectromechanical systems have become widespread within the consumer market, driven by the importance of final product pricing. Presently, MEMS are used in almost every aspect of everyday human life: robotics [1], medical applications [2], transportation [3], geology [4], gaming industry [5], and athletic pursuits [6].

Further, MEMS technology has simplified the incorporation of accelerometers, granting its applicability in vehicles [7], smart watches [8], smartphones [9], gamepads [10], quadcopters [11], and a myriad other devices and systems. Traditional micromechanical accelerometers (MMAs) integrate an elastic suspension within their structure, which contributes to their vulnerability to vibration and impact. Consequently, their application sphere is limited. These limitations might be overcame by introducing MMA based on surface acoustic waves (SAWs). By virtue of the rigid attachment mechanism employed in SAW-based MMA, these devices exhibit a notably enhanced capacity to survive significant overloads compared to conventional MEMS. In addition to the merits shared with conventional MMA, this category of devices also has the advantages in the form of stability and reliability of parameters, repeatability of characteristics, and the possibility of wireless operation [12].

Sensors on surface acoustic waves have found extensive application in multiple areas, serving tasks from magnetic field analysis [13] and temperature regulation [14] to gas analysis [15] and vibration management [16]. The realm of devices founded on acoustic waves presents a promising avenue, with nearly limitless potential for sensor construction variations [17,18,19].

Our research is directed at refining the design of rectangular and triangular sensitive elements (SEs) for micromechanical accelerometers on surface acoustic waves. The current configuration suffers from the drawback of the unilateral attachment of the piezoelectric element to the sensor case, resulting in uneven load distribution. Previously, we proposed an SAW-based micromechanical accelerometer design employing a ring-shaped sensitive element [20]. Investigations have encompassed fixation mechanisms within the sensor housing, determination of the sensitive element’s specific gravity, evaluation of frequency characteristics, and analysis of the impact of external factors, such as excessive acceleration and temperature, on the sensitive element [21]. Additionally, we explored diverse topologies of interdigital transducer (IDT) structures [22].

The focus of this study lies in assessing the optimal overall dimensions of the sensitive element, considering technical constraints and capabilities for subsequent precise measurements. Of particular interest is the assessment of the maximum permissible acceleration for the sensitive element of a ring resonator depending on its parameters, since in the considered design of the sensitive element there are no moving elements (unlike traditional designs of microaccelerometers). Furthermore, this study involves a comparative analysis of the characteristics observed in experimental samples with the data obtained through modeling.

## 2. Design of the Sensitive Element

**Figure 1:**
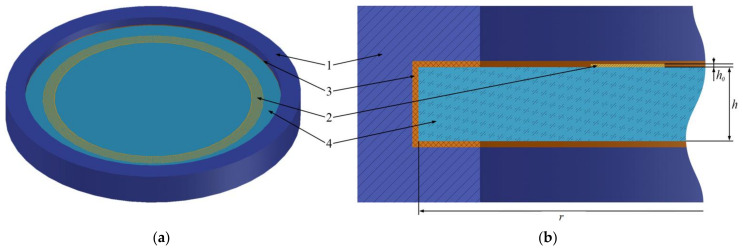
The design of the sensitive element based on surface acoustic waves. General view (**a**) and front view (**b**): 1: case; 2: interdigital transducer; 3: silicone adhesive; 4: substrate.

The general view of the sensitive element of the ring resonator is used from [21] with the substrate attached to the case with silicone glue (Figure 1). The resonator consists of two circular interdigital transducers in the form of trapezoids (2) and a piezoelectric crystal (4) located between the transducers (4). The piezoelectric crystal is fixed in case (1) using silicone adhesive (3). The entire structure is limited both in depth and radius by a damping medium to suppress parasitic wave reflections from external boundaries.

**Figure 2:**
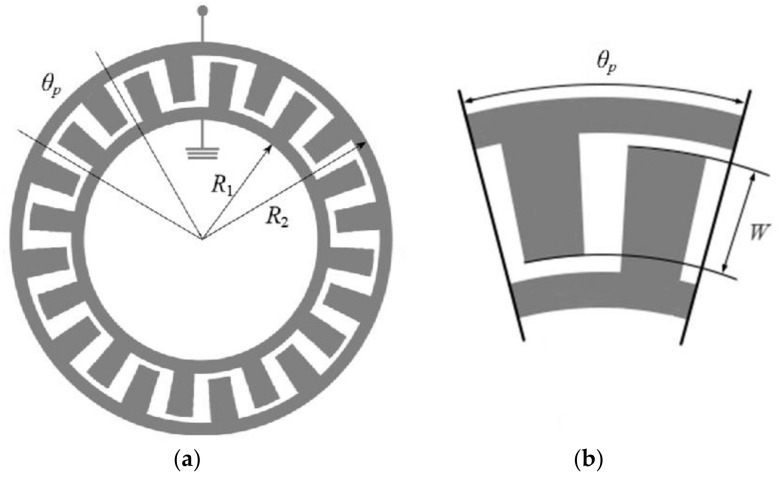
General shape of an interdigital transducer with trapezoidal electrodes (**a**) and a fragment of one angular period (**b**).

The general shape of an interdigital transducer with trapezoidal electrodes is shown in Figure 2. The following IDT parameters are used in this work: the length of the IDT period at the outer edge of the aperture is 19.2 µm for the first case, 38.4 µm for the second, and 57.6 µm for the third at an angular period of the transducer *θ_p_* = 1° and height *h*_0_ = 0.2 µm.

The thickness of the plate according to the technical characteristics of the lithium niobate substrates used to fabricate experimental samples is 350 microns. The plate overhang is 1500 µm for the first case, 3000 µm for the second case, and 4500 µm for the third case. The IDT is located [21] at 1000, 2000, and 3000 µm from the console center for each version, respectively. The use of several options for the overall parameters of the IDT and the substrate is due to the fact that with an increase in the surface area, the substrate will deform less and, accordingly, the sensor will have a lower sensitivity. One of the goals of this work is to find the optimal overall parameter of the ring resonator in relation to the sensitivity of the sensor. The overall parameters of the sensitive elements studied in the work are presented in Table 1. The characteristics of the used materials are presented in Table 2, Table 3, Table 4 and Table 5 [23,24].

## 3. Computer Modeling

COMSOL Multiphysics software version 5.6 was used to simulate physical processes. The grid spacing was chosen as 1/20 wavelength. The number of elements after applying the mesh to the model was more than 25 million. The simulation was carried out on a server with the following characteristics: Intel Gold 6530 (32 × 2.1 GHz), 1024 GB DDR5 ECC and 3× NVIDIA A2.

**Figure 3:**
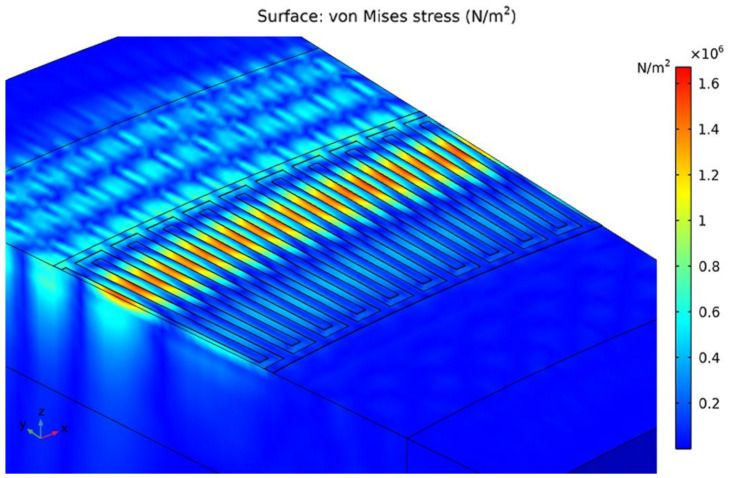
Fragment of a model of a sensitive element with an internal radius of 2000 μm.

**Figure 4:**
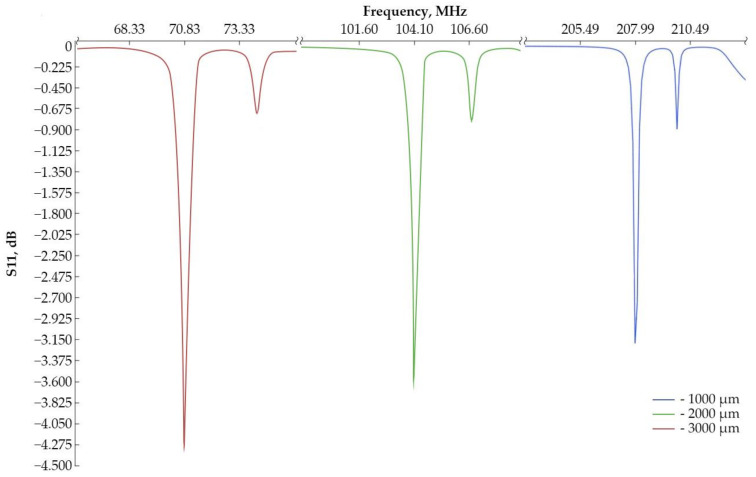
Amplitude–frequency characteristic of the IDT.

At the first stage, it is required to determine the frequency characteristics for models with an inner radius of the IDT of 2,000 µm and 3,000 µm for a subsequent comparison of the obtained data with experimental samples. Figure 3 shows a fragment of a sensitive element with 10 IDT periods and the propagation of a surface acoustic wave along the model. Figure 4 shows the frequency characteristics for various types of sensitive element.

After the simulation, we were able to obtain the following data:

− The resonant frequency for the first sample is 207.99 MHz [22], for the second sample, 104.10 MHz, and for the third, 70.83 MHz.
− The wave reflection coefficient from the input (S11): for a console with an internal IDT radius of 1000 µm, the coefficient is −3.152 dB; for 2000 µm, it is −3.551 dB; and for 3000 µm, it is −4.326 dB.

**Figure 5:**
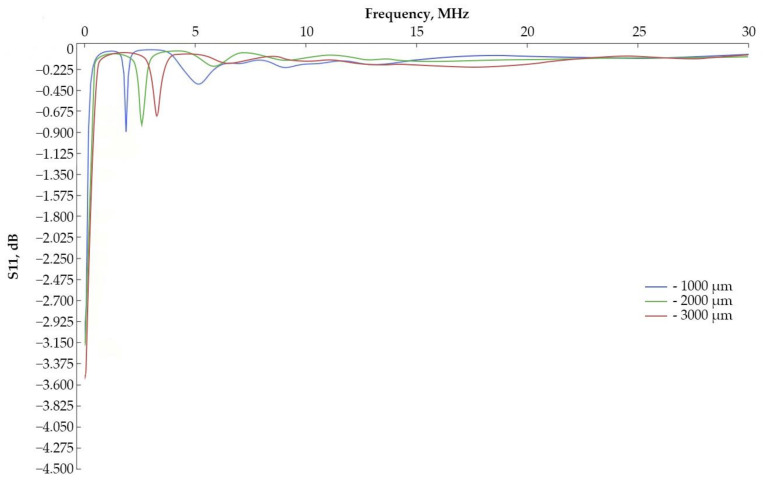
Amplitude–frequency characteristic of the IDT plotted from the resonant frequency.

For a better understanding, graphs of the amplitude–frequency characteristic were plotted relative to the first mode (Figure 5).

It can be concluded that the frequency characteristics of the samples have the same appearance, and the resonant frequency is shifted due to a change in the length of the IDT period. As can be seen from Figure 4 and Figure 5, the wave reflection coefficient from the input increases with increasing dimensions of the sensitive element. Also, the second mode shifts as the console radius increases, and its peak decreases.

**Figure 6:**
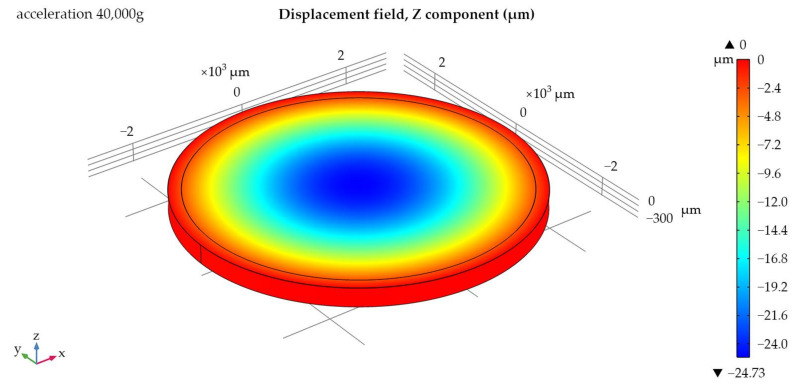
Displacement of the substrate along the z-axis for an IDT with an internal radius of 2000 µm.

**Figure 7:**
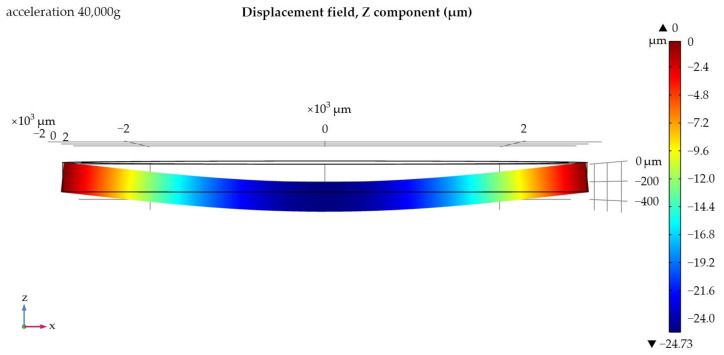
Displacement of the substrate along the z-axis for an IDT with an internal radius of 2000 µm. Diametrical cut, front view.

**Figure 8:**
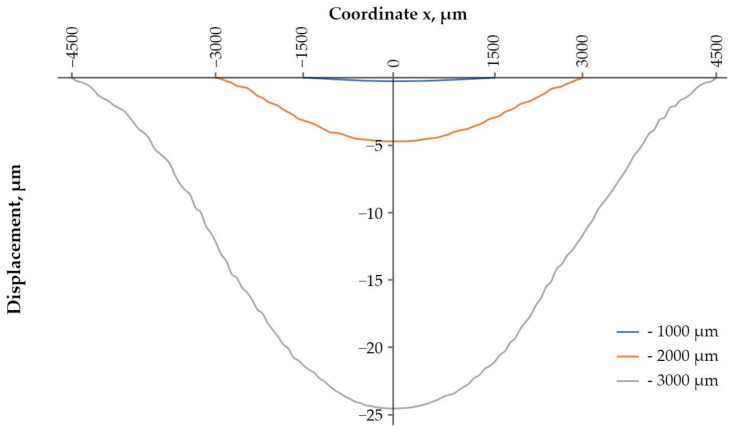
Graph of load distribution along the diametrical section of the substrate at an acceleration of 40,000 g.

**Figure 9:**
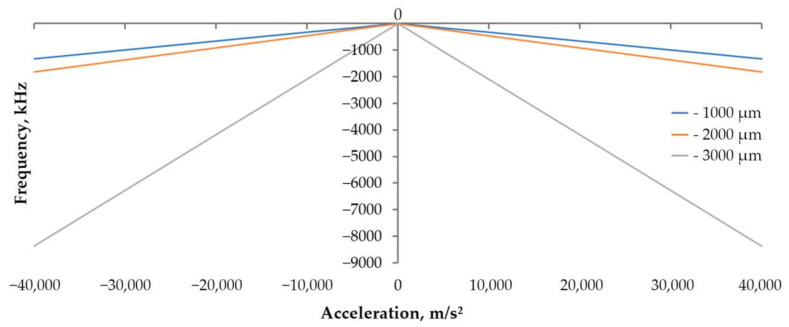
Graph of the frequency change under acceleration.

The next stage is the determination of the maximum permissible acceleration and the sensitivity of each sample. In previous works [21,22], the substrate thickness was taken to be the value at which the SAWs distributed over the bases of the substrate did not interact with each other (7–8 wavelengths). Now, the production of experimental samples will be carried out on a lithium niobate substrate with a thickness of 350 µm; therefore, in order to be able to compare the data, it is necessary to re-simulate with new parameters. Figure 6 and Figure 7 show the displacement of the substrate along the z-axis for one of the samples; Figure 8 shows the graphs of the load distribution along the diametrical section of the substrate for three samples; and Figure 9 shows the graph of the change in frequency from acceleration for three samples. To construct this graph, the following formula was used (1):

(1) $$f=f_{0}\cdot \left(1+\frac{m\cdot r^{2}}{\pi \cdot h^{2}\cdot E}\cdot \chi_{\varepsilon }\cdot a\right)$$

where *f*—frequency of the sensitive element when exposed to acceleration; *f*_0_—frequency of the sensitive element in the absence of acceleration; *m*—weight of the sensitive element with interdigital transducers; *r*—radius of the substrate; *h*—height of the substrate; *E*—young’s module of the substrate material; *χ*_ε_—strain sensitivity coefficient; and *a*—acceleration acting on the sensitive element.

To better perceive the effect of acceleration on the console, in Figure 7, the effect of deformation was visually increased.

**Figure 10:**
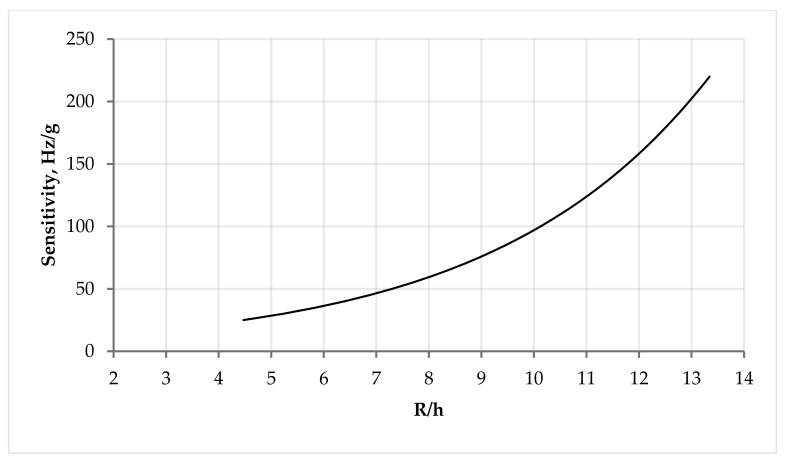
Graph of dependence of sensitivity on the ratio of the radius of the substrate to its thickness.

Based on the data presented in Figure 6, Figure 7, Figure 8 and Figure 9, we can conclude that with the same substrate thickness, the sensitivity of the sensor will increase with an increase in the radius of the substrate. The dependence of sensitivity on the ratio of the plate’s radius to its thickness can be represented as a graph (Figure 10).

**Figure 11:**
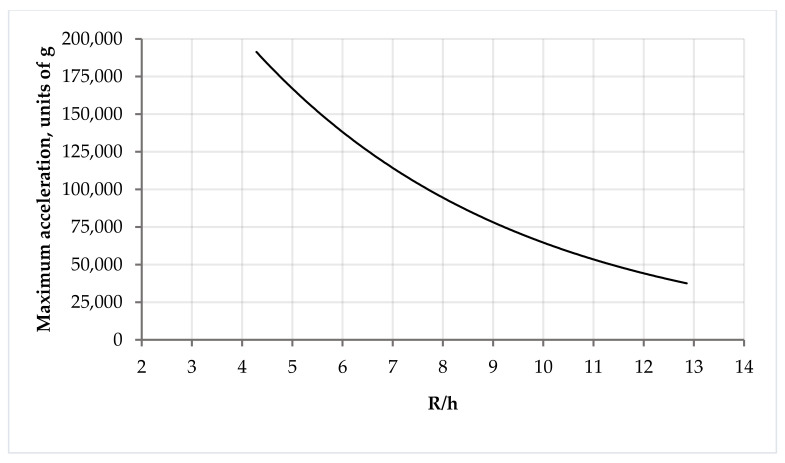
Graph of dependence of maximum acceleration on the ratio of the radius of substrate to its thickness.

The maximum acceleration experienced by the first sample (Table 1) is 191,132 g; by the second sample, it is 84,958 g, and by the third sample, it is 37,514 g. The acceleration that the console is able to withstand depends on the ratio of the radius of the substrate to its thickness, and this dependence can also be represented in the form of a graph (Figure 11).

## 4. Experimental Samples

**Figure 12:**
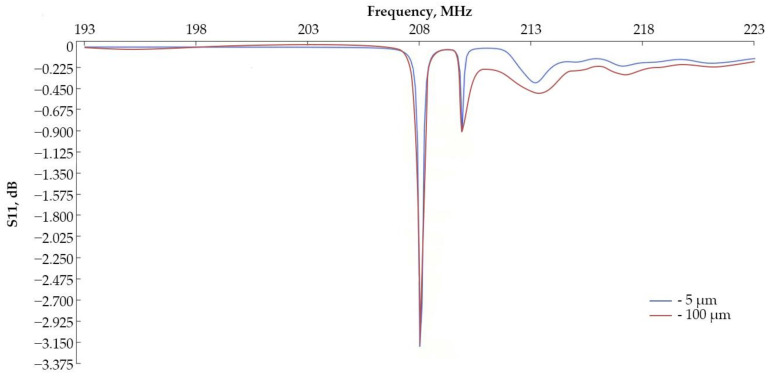
Amplitude–frequency response of IDT depending on bus width.

In order to be able to make experimental samples, according to the manufacturer’s recommendations, the outer and inner bus bars of the IDT were enlarged within a radius of up to 100 µm to be able to connect them to the pad. Additional modeling was carried out to evaluate the effect of enlarged IDT bus bars. The frequency response graph for a sensing element with enlarged IDT buses is shown in Figure 12.

Looking at the simulation results in Figure 12, increasing the bus has little effect on the frequency response of the model. From now on, all characteristics of models and experimental samples coincide.

**Figure 13:**
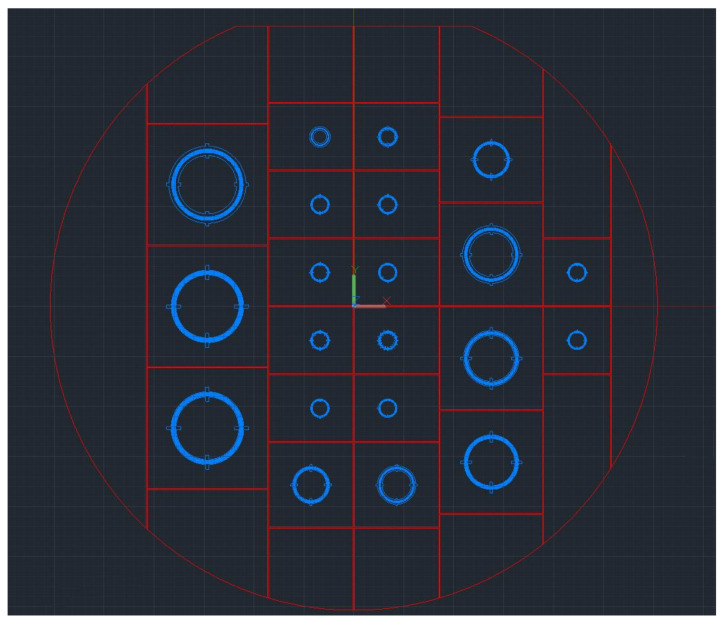
Layout file. Red: plate cut boundaries; blue: interdigital transducers.

All experimental samples were made on a YX-128° cut of lithium niobate with a thickness of 350 µm. As a basis for the manufacture of the sensitive element, the YX-128° cut of lithium niobate plates with a thickness of 350 microns and a diameter of 76 mm were purchased from the enterprise manufacturing the experimental sample. We provided the company with a layout file in .dxf format (Figure 13). In a 1:1 scale file, the interdigital transducers and plate cutting boundaries were optimally placed, taking into account the ability of surface acoustic waves to be reflected from the interfaces.

The topology of aluminum interdigital transducers was created using photolithography. At the end of the etching process and after the surfaces were cleared of photoresist, the plate was cut according to the layout file and its fragments were placed on circuit boards. Next, the thinnest wires were soldered at the base of the circuit board and at the 100 × 100 micron areas of the common buses of the interdigital transducers. This operation was performed using a microscope.

**Figure 14:**
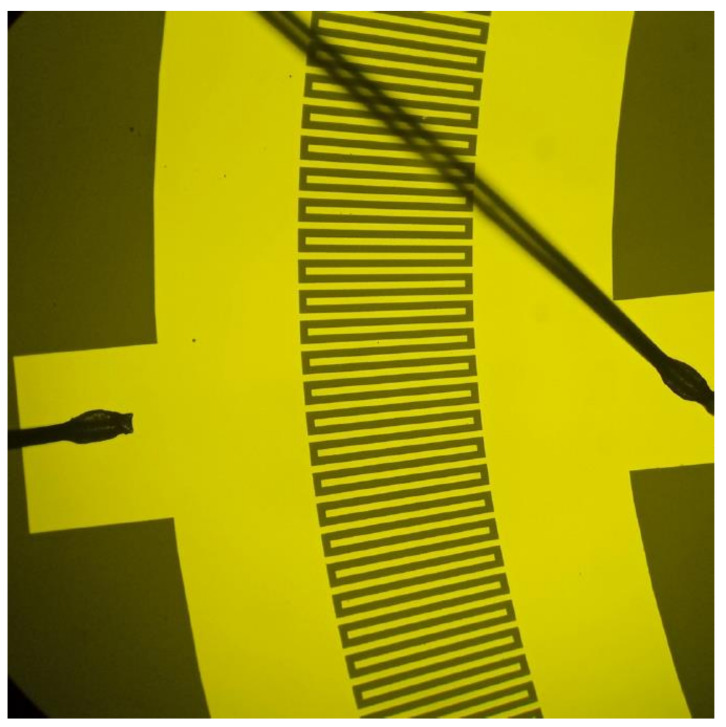
IDT structure under a microscope.

Figure 14 shows a photograph of the experimental sample under a microscope.

**Figure 15:**
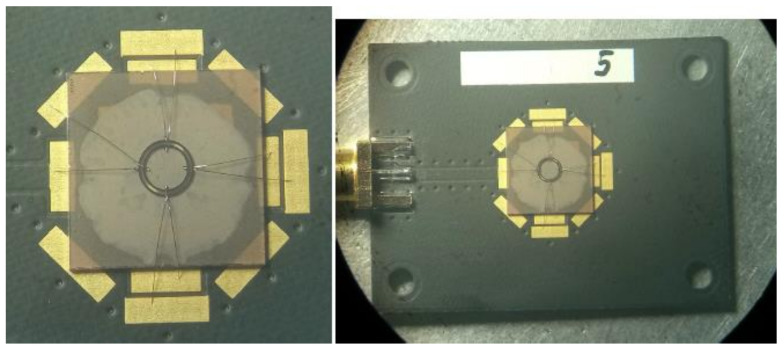
A sample of a ring resonator with an inner radius of the IDT of 1000 μm.

Figure 15 shows a sample of a ring resonator with an IDT inner radius of 1000 µm. To measure the reflective characteristics, the experimental sample was connected to a vector network analyzer Obzor-TR1300/1 [25], which in turn was connected to a PC with specialized software for working with Obzor-TR1300/1.

**Figure 16:**
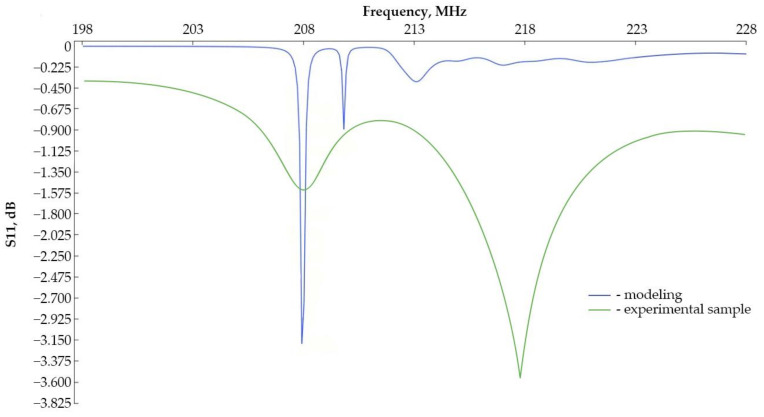
Amplitude–frequency response for a ring resonator with an internal IDT radius of 1000 μm.

Based on the results in Figure 16, we can say that the model built earlier is adequate, since the graphs have the same form, and the resonance frequencies of the model (207.99 MHz) and the experimental sample (218.17 MHz) differ by less than 5%. The formation of the second harmonic in the vicinity of the resonance frequency is associated with an incorrect way of fixing the sensitive element.

Samples of a ring resonator with an IDT inner radius of 2000 and 3000 µm were also fabricated. The data obtained are presented in Table 6.

As can be seen from the results presented in Table 6, although the resonance frequency obtained by modeling and testing the samples is the same, the values of the bandwidth and quality factor are very different. This is due to many factors: the computer simulation is idealized, the experimental sample did not have impedance matching, the signal was not filtered, and aluminum oxidation occurred because the sensitive element was not packaged.

Currently, an electrical circuit is being developed for an experimental sample of a sensitive element of a micromechanical accelerometer based on surface acoustic waves.

## 5. Conclusions

This paper describes further steps in the development of an SAW-based accelerometer utilizing a circular IDT structure. Samples with inner circle radii of 1000, 2000, and 3000 µm were firstly simulated and then fabricated and tested. Discrepancy of the central frequency between the simulation and experiment was about 5% for all three cases, showing a satisfactory model behavior.

The wave reflection coefficient from the input (s11) for the studied samples was from –3.152 dB to −4.326 dB. It shows that all the samples work correctly as SAW resonators and can be used as an accelerometer sensitive element.

The sensitivity and maximum-allowed acceleration of the sensitive element of the ring resonator on surface acoustic waves directly depend on the ratio of the substrate radius to its height, and the higher the sensitivity, the lower the maximum-allowed acceleration. For each material, these dependencies are unique. As an example, maximum acceleration for the first sample (according to Table 1) is 191,132 g; for the second sample, it is 84,958 g; and for the third sample, it is 37,514 g.

This article expands the previously presented knowledge about ring resonators on surface acoustic waves for the sensitive elements of micromechanical accelerometers:

− Scaling the size of the resonator allows us to increase the depth of the frequency peak. However, maintaining the bandwidth reduces the quality factor of the resonator.
− The radial size of the common power bus does not affect the amplitude–frequency response of the ring resonator.

## Figures and Tables

**Table 1:** Dimensions of sensitive elements.

Parameter	Value		
Sample	1	2	3
*R_1_* (inner radius)	1000 µm	2000 µm	3000 µm
*R_2_* (outer radius)	1120 µm	2240 µm	3360 µm
*W* (aperture)	102 µm	204 µm	306 µm
*θ_ext_* (period length on the outer part of the aperture)	19.2 µm	38.4 µm	57.6 µm
*θ_p_* (angular period)	1°		
*h_0_* (IDT thickness)	0.2 µm		
*r* (substrate radius)	1500 µm	3000 µm	4500 µm
*h* (substrate height)	350 µm		

**Table 2:** Characteristics of piezoelectric materials and silicone adhesive.

Parameter	YX-128°LiNbO_3_	Silicone Adhesive
Wave velocity, *ϑ_p_* [m/s]	3961	–
Density, *ρ* [kg/m^3^]	4640	1700
Elastic modulus, *E* [Pa]	170 × 10^9^	25 × 10^6^
Poisson’s ratio, *υ*	0.25	0.48
Tensile strength [Pa]	110 × 10^6^	–

**Table 3:** Matrix form of the tensor of elasticity of the 4th rank of the cut YX-128° of lithium niobate (GPa).

	${c_{E}}_{m1}$	${c_{E}}_{m2}$	${c_{E}}_{m3}$	${c_{E}}_{m4}$	${c_{E}}_{m5}$	${c_{E}}_{m6}$
${c_{E}}_{1n}$	202.900	69.985	57.842	12.846	0	0
${c_{E}}_{2n}$	69.985	193.970	90.330	9.312	0	0
${c_{E}}_{3n}$	57.842	90.330	221.160	8.003	0	0
${c_{E}}_{4n}$	12.846	9.312	8.003	75.323	0	0
${c_{E}}_{5n}$	0	0	0	0	56.860	−5.092
${c_{E}}_{6n}$	0	0	0	0	−5.092	77.919

**Table 4:** Coupling matrix cut YX-128°of lithium niobate, C/m^2^.

	$e_{m1}$	$e_{m2}$	$e_{m3}$	$e_{m4}$	$e_{m5}$	$e_{m6}$
$e_{1n}$	0	0	0	0	4.4724	0.2788
$e_{2n}$	−1.8805	4.4467	−1.5221	0.0674	0	0
$e_{3n}$	1.7149	−2.6921	2.3136	0.6338	0	0

**Table 5:** YX-128°lithium niobate relative permittivity matrix.

	$\epsilon_{{rS}_{m1}}$	$\epsilon_{{rS}_{m2}}$	$\epsilon_{{rS}_{m3}}$
$\epsilon_{{rS}_{1n}}$	0	0	0
$\epsilon_{{rS}_{2n}}$	−1.8805	4.4467	−1.5221
$\epsilon_{{rS}_{3n}}$	1.7149	−2.6921	2.3136

**Table 6:** Comparison of resonance frequencies of models and experimental samples.

		1000 µm	2000 µm	3000 µm
**Model**	Resonance frequency	207.99 MHz	104.10 MHz	70.83 MHz
	Bandwidth	184 kHz	172 kHz	167 kHz
	Q-factor	1130.32	605.27	424.17
**Sample**	Resonance frequency	218.17 MHz	109.23 MHz	74.61 MHz
	Bandwidth	1658 kHz	1531 kHz	1472 kHz
	Q-factor	131.57	71.35	50.64

## Data Availability

The data presented in this study are available on request from the corresponding author.
